# Overexpression of *BdMATE* Gene Improves Aluminum Tolerance in *Setaria viridis*

**DOI:** 10.3389/fpls.2017.00865

**Published:** 2017-06-08

**Authors:** Ana P. Ribeiro, Wagner R. de Souza, Polyana K. Martins, Felipe Vinecky, Karoline E. Duarte, Marcos F. Basso, Bárbara A. D. B. da Cunha, Raquel B. Campanha, Patrícia A. de Oliveira, Danilo C. Centeno, Geraldo M. A. Cançado, Jurandir V. de Magalhães, Carlos A. F. de Sousa, Alan C. Andrade, Adilson K. Kobayashi, Hugo B. C. Molinari

**Affiliations:** ^1^Genetics and Biotechnology Laboratory, Embrapa AgroenergyBrasilia, Brazil; ^2^Plant Biotechnology Program, Federal University of LavrasLavras, Brazil; ^3^Biomass and Biofuels Chemistry Laboratory, Embrapa AgroenergyBrasilia, Brazil; ^4^Centre of Natural Sciences and Humanities, Federal University of ABCSão Bernardo do Campo, Brazil; ^5^Center of Genetic Engineering and Molecular Biology, Embrapa GenClima, University of Campinas, CampinasBrazil; ^6^Applied Biology Center, Embrapa Maize and SorghumSete Lagoas, Brazil; ^7^INOVACAFÉ, Embrapa CoffeeLavras, Brazil

**Keywords:** aluminum, *Setaria viridis*, *MATE*, abiotic stress, hydroponic system

## Abstract

Acidic soils are distributed worldwide, predominantly in tropical and subtropical areas, reaching around 50% of the arable soil. This type of soil strongly reduces crop production, mainly because of the presence of aluminum, which has its solubility increased at low pH levels. A well-known physiological mechanism used by plants to cope with Al stress involves activation of membrane transporters responsible for organic acid anions secretion from the root apex to the rhizosphere, which chelate Al, preventing its absorption by roots. In sorghum, a membrane transporter gene belonging to multidrug and toxic compound extrusion (MATE) family was identified and characterized as an aluminum-activated citrate transporter gene responsible for Al tolerance in this crop. *Setaria viridis* is an emerging model for C4 species and it is an important model to validate some genes for further C4 crops transformation, such as sugarcane, maize, and wheat. In the present work, *Setaria viridis* was used as a model plant to overexpress a newly identified MATE gene from *Brachypodium distachyon* (*BdMATE*), closely related to *SbMATE*, for aluminum tolerance assays. Transgenic *S. viridis* plants overexpressing a *BdMATE* presented an improved Al tolerance phenotype, characterized by sustained root growth and exclusion of aluminum from the root apex in transgenic plants, as confirmed by hematoxylin assay. In addition, transgenic plants showed higher root citrate exudation into the rhizosphere, suggesting that Al tolerance improvement in these plants could be related to the chelation of the metal by the organic acid anion. These results suggest that *BdMATE* gene can be used to transform C4 crops of economic importance with improved aluminum tolerance.

## Introduction

Acidic soils are widely distributed worldwide, predominantly in tropical and subtropical areas, reaching around 50% of the arable soil in the world (Von Uexküll and Mutert, 1995; Cançado, 2006; Kochian et al., 2015). This type of soil strongly reduces crop production, mainly because of the presence of aluminum therein. Aluminum (Al) is the most abundant metal on Earth, and conjugated with oxides, silicates, and hydroxides constitutes soil particles (Boff, 2006; Oliveira, 2012). In highly acidic soils (pH < 5), Al is solubilized into a phytotoxic form Al(H_2_O)_6_^3+^, usually referred to as Al^3+^. Aluminum is extremely rhizotoxic, affecting root growth and function and therefore limiting crop production and its toxicity, along with drought, is a primary food security concern (Von Uexküll and Mutert, 1995; Magalhães et al., 2007).

A well-known physiological mechanism used by plants to cope with Al stress involves the activation of plasma membrane transporters responsible for organic acid anions secretion from the root apex to the rhizosphere (Kochian et al., 2015). These anions form non-phytotoxic stable complexes with Al^3+^, preventing its absorption by the roots (Boff, 2006; Kochian et al., 2015). In sorghum (*Sorghum bicolor*), a membrane transporter gene belonging to the *MATE* family was identified and characterized as an aluminum-activated citrate transporter gene responsible for the Al tolerance in this crop, codifying for a proton-dependent transporter protein (Magalhães et al., 2007). *Arabidopsis thaliana* plants overexpressing the *S. bicolor MATE* gene (*SbMATE*) demonstrated an Al tolerant phenotype and increased root citrate exudation when compared to NT plants (Magalhães et al., 2007). These results support that a member of a *MATE* family from *S. bicolor* (*Sb*MATE) is an Al-activated citrate secretion transporter conferring aluminum tolerance. In addition, it was demonstrated that MATE homologs are root citrate transporters in other monocot species such as maize (*Zm*MATE1, Maron et al., 2010, 2013), and rice (*Os*FRD1, Yokosho et al., 2011). Moreover, the expression of the citrate transporters *SbMATE* and *FDR3* in barley enhanced the Al tolerance in this sensitive cereal (Zhou et al., 2014). Other study demonstrated that a *MATE* gene cloned and isolated from *Vigna umbellata* (*VuMATE*), when expressed in tomato, was able to increase Al resistance, accompanied by enhanced citrate efflux (Yang et al., 2011). Currently, some physiological and molecular mechanisms of organic acid anions exudation are described. Several studies have implicated that plasma membrane H^+^-ATPases are involved in Al tolerance mechanism by citrate exudation. For instance, in soybean and broad bean, the citrate exudation from the roots was associated with the up-regulation of the plasma membrane H^+^-ATPase, indicating that this protein modulates Al-induced citrate exudation (Shen et al., 2005; Chen et al., 2013, 2015). Wang et al. (2016) demonstrated that auxin is possibly involved in Al stress tolerance in soybean, since the concentration of IAA was increased by Al and exogenous application of IAA decreased Al concentration in roots by increasing citrate exudation through upregulation of *GmMATE* and activation of the plasma membrane H^+^-ATPase. In addition to the well described *SbMATE*, a *Brassica oleracea* MATE gene has also been shown to be involved in Al tolerance responses in *A. thaliana*. Transgenic Arabidopsis lines overexpressing *BoMATE* demonstrated Al tolerance and increased citrate exudation. Furthermore, these lines showed a lower K^+^ efflux and higher H^+^ efflux in the presence of Al, indicating that MATE is involved in the K^+^ and H^+^ fluxes during Al stress (Wu et al., 2014). Therefore, MATE, along with the anion channels ALMTs (Sasaki et al., 2004), are potential root organic acid anion transporters involved in Al resistance in plants and are potential targets for crop improvement under Al stress.

*Setaria viridis* is an emerging monocot plant model for molecular and genetic studies (Muthamilarasan and Prasad, 2015). It is a short, fast-growing, C4 plant with its genome sequence fully available (Bennetzen et al., 2012), making it a reliable model for genetic studies. In addition, *S. viridis* is amenable for genetic transformation through *Agrobacterium tumefaciens*, with well-established transformation protocol (Martins et al., 2015). In the present work, *S. viridis* was used as a model plant system to overexpress a new ortholog of *Sorghum bicolor MATE* (*SbMATE*), *Brachypodium distachyon MATE* (*BdMATE*), under the control of the maize ubiquitin constitutive promoter (*Zm*Ubi-1) for aluminum tolerance. The *BdMATE* gene was chosen because it is up-regulated under Al conditions (Contreras et al., 2014), whereas *S. viridis MATE* is not (this study, data not shown). The rationale was to use a close homolog of *SbMATE* and a patent-free gene, in order to use as proof of concept to further transform C4 crops of economic importance such as sugarcane, wheat, and maize, which are difficult and time-consuming to transform. *S. viridis* plants overexpressing *BdMATE* showed an Al tolerance phenotype, characterized by sustained root growth under {20} μM Al^3+^, whereas NT plants showed RGI. Moreover, exclusion of aluminum from the root apex in transgenic plants was inferred based on results from hematoxylin assays. In addition, transgenic plants constitutively overexpressing *BdMATE* presented higher citrate secretion levels when compared to NT plants under Al stress, suggesting that the mechanism of organic acid anion exudation and consequent Al chelation by these compounds are involved in the Al toxicity amelioration in these plants. The expression of aluminum tolerance-related genes was evaluated, and the results obtained are discussed based on the known Al tolerance. Our results demonstrated that, in the future, the overexpression of *BdMATE* could be used to develop Al-tolerant crops closely related to *S. viridis*, such as maize, wheat, and sugarcane.

## Materials and Methods

### *In Silico* Analysis

Searches for the *Sorghum bicolor MATE* (*Sb*MATE) gene in the Phytozome database were performed in order to find orthologs in the genome of *B. distachyon*. Based on the different sequences found, the alignment of these sequences was generated using the Clustal Omega (Sievers and Higgins, 2014). Analysis of conserved protein domains and the prediction of the molecular and cellular functions of these domains were evaluated by the online softwares FFPred and MEMSAT (Nugent and Jones, 2009; Buchan et al., 2010). The phylogenetic tree was generated based on amino acid sequences of major grasses (Contreras et al., 2014) that have been studied with *MATE* gene, using the Geneious software (Kearse et al., 2012) for alignments and MEGA7 software (Kumar et al., 2016) for building phylogenetic tree.

### Tissue Culture and Plant Transformation

In the present work, we were willing to validate an orthologous of the *Sorghum bicolor MATE* (*SbMATE*) gene that confers aluminum tolerance in different species (Magalhães et al., 2007; Sivaguru et al., 2013). The gene chosen was *BdMATE*, a close homolog of *SbMATE* from *B. distachyon*, a model organism for grasses (Kellogg, 2015), also studied by our group. The gene construct containing the *B. distachyon MATE* gene (*BdMATE*), driven by *Zm*Ubi1 constitutive promoter and the *hpt* gene selection marker (which confers hygromycin resistance), was synthetized by DNA Cloning Service, Germany (Supplementary Figure S1) and introduced into *A. tumefaciens* strain EHA105 for plant transformation.

Mature seeds of *S. viridis* accession A10.1 were selected for embryogenic calli induction in CIM [consisted of MS salts (Murashige and Skoog, 1962), 1 mg/L d-biotin, 0.5 mg/L pyridoxine HCl, 0.5 mg/L nicotinic acid, 100 mg/L myo-inositol, 0.1 mg/L thiamine-HCl, 0.6 mg/L CuSO_4_, 30 g/L sucrose, 2 mg/L 2,4-dichlorophenoxyacetic acid, 0.5 mg/L kinetin, and 4 g/L Phytagel^TM^, pH 5.8]. Induced calli were used for *Agrobacterium*-mediated transformation, according to Martins et al. (2015). The putative transgenic calli, resistant to 30 mg/L hygromycin B, were transferred to SRM consisting of MS salts (Murashige and Skoog, 1962), 1 mg/L d-biotin, 0.5 mg/L pyridoxine HCl, 0.5 mg/L nicotinic acid, 100 mg/L myo-inositol, 0.1 mg/L thiamine HCl, 20 g/L sucrose, 2 mg/L kinetin, 150 mg/L Timentin^^®^^, 30 mg/L hygromycin B, 2 g/L Phytagel^TM^, pH 5.8. The regenerated plants were submitted to PCR analysis in order to confirm the presence of the transgene.

### Molecular Analysis and Selection of the Transgenic Events

Genomic DNA from regenerated plantlets resistant to hygromycin was extracted using a modified CTAB method (Doyle and Doyle, 1990), according to Molinari et al. (2007). The gene insertion was confirmed by PCR using specific primers designed for *BdMATE* amplification (Supplementary Table S1).

The putative transgenic events underwent gene expression analysis by real-time PCR of the target gene *BdMATE*, using the primers described in Supplementary Table S1. The expression level was calculated using endogenous genes *SiUBC* e *SiSDH2* as reference (Martins et al., 2016).

Segregation analysis was performed in *T*_1_ seeds to estimate the number of insertions in each event. Seeds were grown in selective medium with hygromycin B 50 mg/L. After 7 days, the number of resistant and sensitive plants was counted, and the proportion was analyzed statistically using the χ-Squared test. Events with single insertion determined by 3:1 Mendelian segregation ratio were selected. The *T*_2_ seeds of the selected events were placed in selective medium with hygromycin B 50 mg/L, for the selection of transgenic homozygous lines, which were used for aluminum stress assays.

### Aluminum Treatment Assay and Root Growth Measurement

A protocol was established in order to evaluate the phenotype of *S. viridis* submitted to aluminum treatment in hydroponic system. First, NT 7-days-old plantlets were subjected to evaluation of root growth in different nutrient solutions such as Hoagland (Hoagland and Arnon, 1950), Camargo (Camargo, 1981), and Magnavaca (Magnacava et al., 1987) (Supplementary Figure S2). The growth of the plants under a solution based solely on CaCl_2_ (calcium chloride), at different concentrations, (Supplementary Figure S3) was also tested, according to Sasaki et al. (2004). The normal plant growth verified under 500 μM CaCl_2_ solution prompted us to use it for our studies. Al^3+^ activity was estimated using the software GeoChem-EZ (Shaff et al., 2010) (**Table 1** and Supplementary Figure S4). After the establishment of the stress protocol, a solution containing {20} μM of Al^3+^ free activity, pH 4.2, was used to submit *S. viridis* plants to Al^3+^ for 1, 3, and 5 days, and this protocol was followed throughout the work. The evaluation of the root growth was performed by carefully scanning the roots daily for 5 days, in order not to damage the tissue, and the total root length was measured by the software WinRhizo 2007a (Régent Instruments). Transgenic and NT plants were evaluated for liquid root growth, and the percentage of RGI in treatments in the presence or absence of aluminum was calculated as described in Ryan et al. (2009). In order to measure RNG, the length of the roots was measured before and after each day of growth in the calcium chloride solution with (+Al) and without (-Al) aluminum. The % RGI values were calculated from root growth measured over each day in +Al solution divided by root growth measured over each day in control (-Al) solution × 100, calculated individually for each plant.

**Table 1:** Concentration and free activity of aluminum in 500 μM CaCl_2_.2H_2_O, pH 4.2, calculated using the software GeoChem-EZ (Shaff et al., 2010).

Concentrations of AlCl_3_	Free activity of Al^3+^
0 μM	{0} μM
18 μM	{10} μM
35 μM	{20} μM

*Values inside the brackets indicate Al^*3+*^ activity estimated with the speciation software, GeoChem-EZ (Shaff et al., 2010)*.

### Real-Time qPCR Analysis

After 7-days-old plantlets had been submitted to 500 μM CaCl_2_ solution, in the absence or the presence of {20} μM Al^3+^, pH 4.2, in hydroponic system for 1, 3, and 5 days, total RNA was extracted from shoots using TRIzol^^®^^ reagent (Thermo Fisher Scientific), according to the manufacturer’s instructions. RNA from roots was extracted using a LiCl method (Chang et al., 1993). The samples were treated with RQ1 RNase-free DNase (Promega, Madison, WI, USA), according to the manufacturer’s instructions. Total RNA was quantified using a NanoDrop ND-1000 Spectrophotometer (Uniscience), and RNA integrity was verified in agarose gel electrophoresis. The synthesis of first strand cDNA was accomplished using the extracted RNA as template and the RevertAid^TM^ First Strand cDNA Synthesis kit (Thermo Fisher Scientific). These steps were all performed according to the manufacturer’s instructions. The qPCR was carried out using Platinum^^®^^ SYBR^^®^^ Green PCR SuperMix-UDG with ROX (Invitrogen, Carlsberg, CA, USA) with synthesized single-stranded cDNA as template, using the protocol recommended by the StepOnePlusReal-Time PCR Systems (Applied Biosystems). The primers used in the qPCR are described in Supplementary Table S1.

Relative gene expression levels were calculated using the q-Gene (Muller et al., 2002). *SiEF1-α*; *SiSUI*, *SiCAC*, and *SiCUL* were used as reference genes (Martins et al., 2016) and the geometrical mean of the relative quantities (RQs) was calculated using BestKeeper software (Pfaffl et al., 2004). Individual amplification efficiencies were established with LinRegPCR v.2013.0 using a window-of-linearity (Ramakers et al., 2003). The experiment was performed using three biological replicates, with 40 plants each.

### Hematoxylin Staining

The exudation of organic acid anions by the root apex will act chelating aluminum in the rhizosphere, forming a neutral complex, thus preventing the root absorption of part of the Al. Staining is described as one of the best and fastest methods to monitor the location and aluminum distribution in plants (Illéš et al., 2006). The hematoxylin method was used to evaluate Al accumulation in *S. viridis* plants under aluminum treatment, similar to treatment described above carried out for root growth measurement. The protocol was based on Tang et al. (2000). The roots were excised from the plantlets and gently shaken in 2 mL of distilled water for 60 min. The water was replaced by 2 mL of aqueous hematoxylin solution (0.2% hematoxylin and 0.02% potassium iodide, w/v) and agitated for 15 min. Finally, the solution was replaced one more time by 2 mL distilled water, thereby repeating the first step. After staining, the roots were photographed under the stereomicroscopic Leica Model S8APO.

### Determination of Root Citrate Exudation

The transgenic and the NT plants were grown hydroponically as described previously, and root exudates were collected after 3 days of growth on nutrient solution containing {0} or {20} μM Al^3+^. After the exposure period, the roots were washed with 20 mL of 500 μM CaCl_2_ solution for 40 min, under 60 rpm in an orbital shaker. This procedure was repeated three times for each replicate. Each replicate (3 in total) was composed of 40 plantlets bulked together for extraction (120 plantlets in total). The extraction was normalized to the root biomass for each line. The solutions were lyophilized for organic acids analysis on a gas chromatography–mass spectrometry (GC-MS) system (Varian GC 3800 and MS 2000). The lyophilized material were washed in pure methanol and dried under vacuum. To the dried samples were added 100 μL of pyridine and 50 μL of BSTFA (N,O-bis(trimethylsilyl)trifluoroacetamide) + 1% TMCS (trimethylchlorosilane). The analysis of GC/MS was performed according to Centeno et al. (2016) on a 30 m HP5 column with 0.25 mm of diameter and 0.25 μM film thickness (Supelco). Helium was used as the carrier gas at a flow rate of 1 mL min^-1^. The analysis was performed under the following temperature program: 5 min of isothermal heating at 70°C, followed by a 5°C min^-1^ oven temperature ramp to 310°C, and a final 1 min of heating at 310°C. Mass spectra were recorded at 2 scan s^-1^ with a scanning range of 50–600 m/z. Both chromatograms and mass spectra were evaluated using the OpenChrom software (Wenig and Odermatt, 2010). The peaks were identified and quantified in comparison with authentic standards and the NIST 08 Mass Spectral Library.

### Statistical Analysis

Experimental data were analyzed using randomized block design (RBD) with replications for each treatment ({0} and {20} μM Al^3+^). Samples for all analysis were collected at days 1, 3, and 5. Differences among treatments per sample were analyzed using *t*-test, considering *p* < 0.05 as significant.

## Results

### *In Silico* Analysis

**FIGURE 1:**
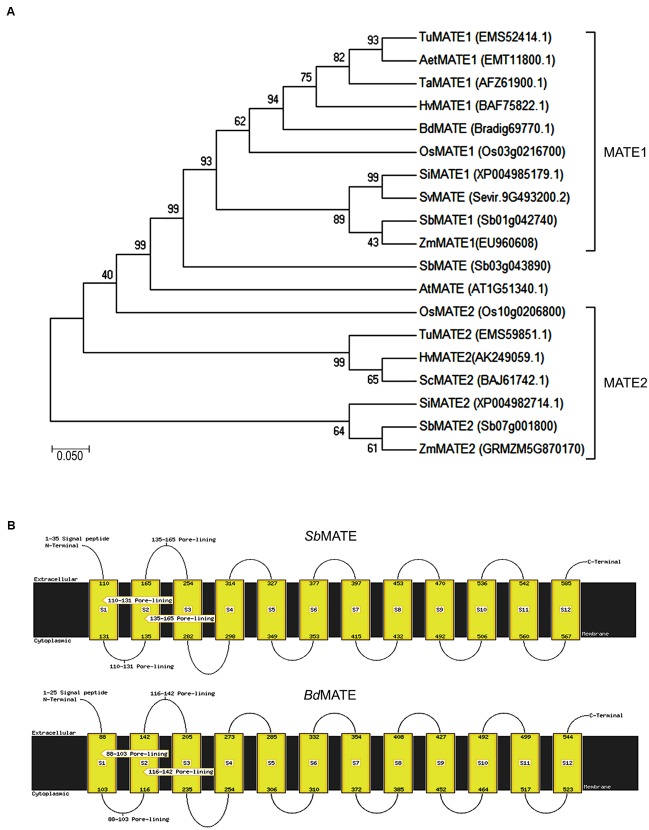
**(A)** Phylogenetic tree based on amino acid sequences. The analyses were conducted in MEGA7 software (Kumar et al., 2016) using the Maximum Likelihood method and bootstrap consensus tree inferred from 10000 replicates. The amino acids sequences of these proteins were obtained from NCBI and Phytozome. **(B)** Transmembrane domains of the *Bd*MATE and *Sb*MATE generated by FFPRED and MEMSAT-SVM (Nugent and Jones, 2009; Buchan et al., 2010).

In order to find orthologous sequences of *S. bicolor MATE* gene, it was conducted a BLAST analysis in the Phytozome database^1^. The hits based on the lowest *E*-values, highest score values and larger alignment bars with the query sequence were chosen for alignment studies using the *MATE* genes present in different organisms. *B. distachyon* (*BdMATE)* gene showed a close similarity to the *SbMATE* gene (Supplementary Figure S5), and therefore *BdMATE* was chosen for further studies. *S. viridis MATE* gene was not chosen because the expression levels of the endogenous *SvMATE* did not change in the presence of Al under our experimental conditions (see below). The selected amino acid sequence of *B. distachyon* MATE protein (*Bd*MATE – Bradi1g69770.1) were aligned using the Geneious software (Kearse et al., 2012) with the different MATE sequences from other related organisms to generate a phylogenetic tree, using MEGA7 software (Kumar et al., 2016) (**Figure 1A**). The sequence Bradi1g69770.1 was chosen for the present study due to its close similarity to the *S. bicolor* MATE sequence.

In addition, *Bd*MATE and *Sb*MATE transmembrane domains analysis was performed, using the softwares FFPRED and MEMSAT-SVM (Nugent and Jones, 2009; Buchan et al., 2010). The results are shown in the **Figure 1B**, where it was demonstrated that the *B. distachyon* MATE possesses the same 12 transmembrane domains presented by the *S. bicolor* MATE protein. *Bd*MATE belongs to the large multifunctional transport family involved in the transport of organic solutes out of the cytoplasm. The putative molecular functions attributed to this protein are transporter activity, ion transmembrane transporter activity, receptor activity, and lipid binding; all these characteristics have shown high specificity and precision for *Bd*MATE, according to MEMSAT (McGuffin et al., 2000; Nugent and Jones, 2009).

### Generation of *BdMATE* Transgenic Lines

After the similarity between *BdMATE* and *SbMATE* has been confirmed by *in silico* analysis, an expression vector containing *Zm*Ubi1::*BdMATE* was constructed in order to transform embryogenic calli of *S. viridis* accession A10.1. Transgenic calli were selected with 30 mg/L of hygromycin B and regenerated under SRM. Genomic DNA was extracted from regenerated plants and used as template for PCR using primers designed for the *BdMATE* gene amplification. The 450 bp amplification product corresponding to the specific region of the *BdMATE* gene was confirmed in the transgenic plants (Supplementary Figure S6A). The gene expression levels were analyzed by qPCR using *BdMATE* specific primers for 30 *T*_0_ transgenic events. Representative events expressing different levels of *BdMATE* were selected for Mendelian segregation analysis. All the selected events showed a 3:1 segregation ratio, indicating a single gene insertion (Supplementary Figure S6B and Table S2).

Subsequently, the events 28 and 29, showing moderate and high expression levels of *BdMATE*, respectively, were chosen to generate homozygous lines. Two different homozygous lines at *T*_3_ generation were obtained, 28.1 and 29.2. The expression of the *BdMATE* gene in these homozygous lines was evaluated in *S. viridis* roots using real-time quantitative PCR (Supplementary Figure S7A) and these lines were chosen for further aluminum tolerance tests.

### Aluminum Tolerance Evaluation in Transgenic Plants

**FIGURE 2:**
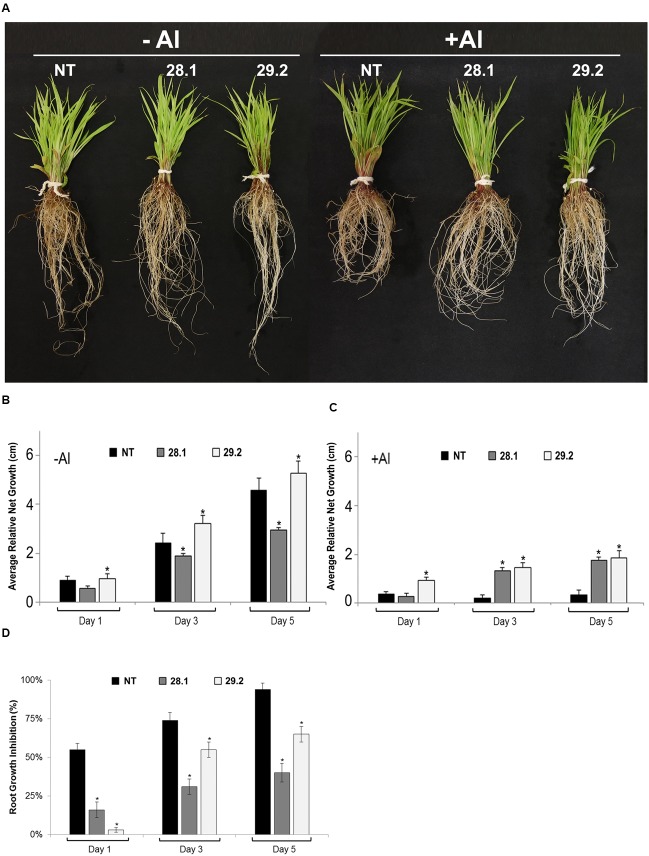
**(A)** *Setaria viridis* homozygous transgenic lines overexpressing *BdMATE* and NT plants in the absence (–Al; left panel) and after 5 days of exposure to {20} μM Al^3+^ (+Al; right panel). **(B,C)** Relative RNG of *S. viridis* homozygous transgenic lines overexpressing *BdMATE* and NT plants grown under the absence (–Al) or presence (+Al) of {20} μM Al^3+^ during 1, 3, and 5 days. The length of the roots was measured before and after each day of growth in the calcium chloride solution with (+Al) and without (–Al) aluminum (*n* = 20 plantlets). **(D)** RGI of *S. viridis* homozygous transgenic lines overexpressing *BdMATE* and NT plants grown under the absence (–Al) or presence (+Al) of {20} μM Al^3+^ during 1, 3, and 5 days. The % RGI values were calculated from root growth measured over each day in +Al solution divided by root growth measured over each day in control (–Al) solution × 100, calculated individually for each plant (*n* = 20 plantlets). ^∗^Significantly different at *P* < 0.05 between –Al and +Al treatments in transgenic lines.

The most common criterion used to measure Al toxicity is the comparison of the root length of Al-affected plants with control plants grown in the absence of aluminum (Ma et al., 1997). In the present study, homozygous *S. viridis* transgenic plants overexpressing the *BdMATE* gene were evaluated by measuring the root growth along 5 days in a hydroponic system in the absence or presence of {20} μM Al^3+^ free activity, in comparison to NT plants. The results demonstrated that transgenic lines presented higher root length compared with NT plants in the presence of Al (**Figure 2A**). In the absence of the metal, both NT plants and the transgenic line 29.2 showed a similar root growth pattern, while the line 28.1 demonstrated a lower root growth even in control conditions (**Figures 2A,B**).

**FIGURE 3:**
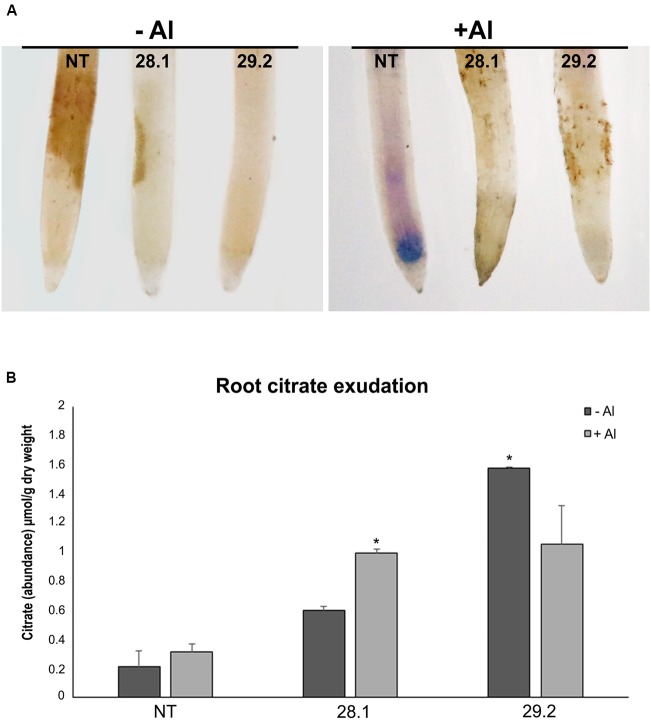
**(A)** Hematoxylin staining after 24 h exposure to {0} and {20} μM Al^3+^ in roots of the *S. viridis* homozygous transgenic lines overexpressing *BdMATE* and NT plant. **(B)** Citrate abundance on roots exudates in the absence and after 3 days of exposure to {20} μM Al^3+^. Citric acid was determined by gas chromatography/mass spectrometry (GC/MS). The values are represented as the mean ± SD. ^∗^Significantly different at *P* < 0.05 between –Al and +Al treatments in transgenic lines.

Quantification of the relative net root growth (RNRG; **Figure 3A**) and the percentage of the RGI (**Figure 3B**) confirmed the Al tolerance phenotype demonstrated by *BdMATE* transgenic plants. It is worth to mention that the morphology of the roots was not affected from the constitutive expression of the *BdMATE* gene. A RGI above 90% was observed in NT plants after 5 days in {20} μM Al^3+^ (**Figure 3B**). Under the same conditions, transgenic plants overexpressing *BdMATE* demonstrated from 30 to 65% of RGI for the events 28.1 and 29.2. According to Magalhães et al. (2007), a plant can be considered tolerant to Al when its RGI during the stress is ≤70%. It is worth to mention that transgenic plants grown in the presence of Al demonstrated a small decrease of root elongation when compared to NT plants, indicating that *BdMATE* overexpression did not fully eliminate the toxic effects of Al, but ameliorates such effect.

### Hematoxylin Staining

Hematoxylin assay was performed after 24 h of plants exposed to {0} or {20} μM Al^3+^. This assay consists of the development of blue-purplish color in areas where aluminum is present in the root tissue. The **Figure 3A** shows accumulation of aluminum in a larger root portion of *S. viridis* NT plants in the presence of Al (+Al), while in the transgenic plants in the same conditions the blue-purplish color representing Al accumulation is absent. The staining of the plants is not observed in plants not submitted to Al (**Figure 3A**; -Al). These results suggest that NT plants are accumulating higher concentrations of Al than transgenic plants, indicating that tolerant plants are avoiding the absorption of Al by the roots.

### Root Citrate Exudation

The measurements of root citrate secretion demonstrated that a new MATE homolog, *BdMATE*, when overexpressed in *S. viridis* plants, was able to increase the levels of this anion in transgenic plants when compared to NT plants (**Figure 3B**). In the presence of {20} μM Al^3+^, the transgenic lines presented a threefold increase of citrate exudation in comparison to the NT plants, suggesting that *BdMATE* plants are conferring Al tolerance through the mechanism of citrate release into the rhizosphere.

### Gene Expression Analysis

#### TCA Cycle Genes

**FIGURE 4:**
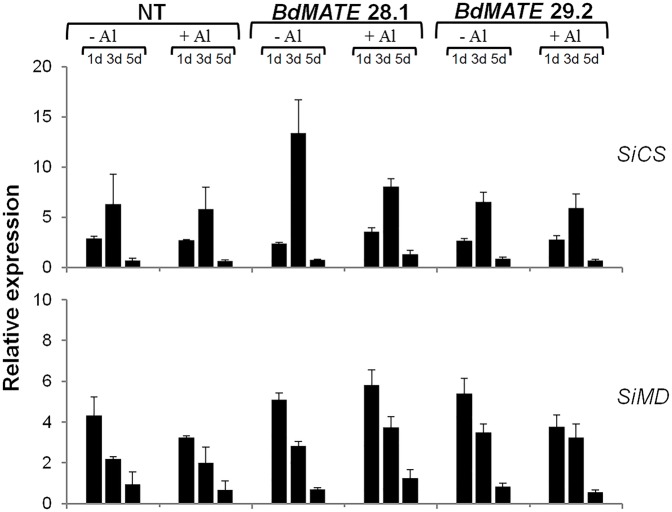
**Relative gene expression levels of the *SiCS* (CS) and *SiMD* (MD) in the *S. viridis* homozygous transgenic lines overexpressing *BdMATE* and NT plants submitted to {0} and {20} μM Al^3+^ during 1, 3, and 5 days**. No significant difference at *P* < 0.05 between –Al and +Al treatments in transgenic lines.

The **Figure 4** demonstrated that no difference of CS (*SiCS*) and MD (*SiMD*) gene expression levels were observed between transgenic and NT plants, in the absence or presence of Al. The signaling of Al tolerance in plants is still a matter of debate and therefore more studies are necessary in order to elucidate the relationship between the levels of citrate produced by the TCA cycle with Al tolerance in plants. The results obtained on gene expression levels will be discussed in the latter section.

#### Expression of Endogenous *S. viridis MATE* Gene

The expression levels of endogenous *S. viridis MATE* gene (Sevir.9g493200.2) were verified to demonstrate the involvement of *SvMATE* in Al stress responses. The Supplementary Figure S7B shows that *SvMATE* expression levels did not change in plants submitted to Al when compared to plants in the absence of the cation, indicating that the endogenous *SvMATE* does not participate in aluminum stress responses in *S. viridis*. Although the similarity of the *MATE* sequence of *S. viridis* and *B. distachyon* is 83.2% (Supplementary Figure S8).

## Discussion

Sugarcane is a very important economic crop for Brazil nowadays. Brazil is the largest sugarcane producer worldwide, and its production is intended mainly for ethanol and sugar production. In addition, the biomass released from the sugarcane process can be used as lignocellulosic material to be degraded by microorganisms to generate renewable fuels and added-value products. Despite of its great importance, the regions for sugarcane growth in Brazil are becoming scarce. An alternative for growing sugarcane could be the Cerrado region, characterized by its soil with acidic pH. Therefore, a great effort has been made by the scientific community in order to improve sugarcane traits for its growth in poor soils. *Setaria viridis* is an emerging model for C4 species and it is an important model to validate some genes for further sugarcane transformation (Muthamilarasan and Prasad, 2015). In the present work, a close homolog of *SbMATE* gene, the *B. distachyon MATE* gene (*BdMATE*) was constitutively overexpressed in *Setaria viridis* (Brutnell et al., 2010), in order to verify aluminum tolerance in transgenic plants. The *BdMATE* gene was chosen instead of the *S. viridis MATE* gene (*SvMATE*) because *BdMATE* is a close homolog of *SbMATE* expressed under Al conditions (Contreras et al., 2014), while *SvMATE* is not (this study, data not shown). The results obtained with the overexpression of *BdMATE* in *S. viridis* plants clearly showed an Al tolerance phenotype in these plants (**Figure 2**). The main symptom of aluminum toxicity is inhibition of root growth, which is observed consistently in plants sensitive to this metal (Kochian et al., 2015). In the presence of Al, the two different homozygous transgenic lines demonstrated higher root length (**Figure 2A**) and RNRG (**Figures 2B,C**) when compared to NT plants. On the other hand, the percentage of RGI of transgenic plants under Al stress was lower when compared to the control (**Figure 2D**). However, it is worth to mention that the root growth of transgenic lines was lower in the presence of Al than in the absence of this metal, indicating that *BdMATE* ameliorates but do not completely eliminates the phytotoxicity effect of this cation.

Furthermore, hematoxylin staining, a common method used to monitor the location and aluminum accumulation in plants (Illéš et al., 2006), demonstrated that *BdMATE* plants did not accumulate Al in the root apex, a phenotype demonstrated by NT plants (**Figure 3B**). The primary site of aluminum accumulation and damage is still a matter of debate. In many species, the root apex was identified as the primary site of perception of aluminum toxicity, and consequently the expression of tolerance (Kollmeier et al., 2000; Yang et al., 2008; Horst et al., 2010; Motoda et al., 2011; Souza et al., 2016). Sivaguru et al. (2013) demonstrated that Al affects approximately the 1–3 mm portion of the roots in sorghum lines sensitive to the metal. Although the effects of aluminum may occur in all parts of the root growing regions, ruptures above pea (Yamamoto et al., 2001), maize (Jones et al., 2006), and bean roots (Kopittke et al., 2008) occurred predominantly in regions within about 1–2 mm of the root tip, in the distal portion known by the DTZ (Sivaguru and Horst, 1998). In order to effectively confer Al resistance via Al-activated citrate exudation, it is logical to assume that the highest density of root citrate transporters should be specifically located in the root regions most affected by Al toxicity (Sivaguru et al., 2013). Our hematoxylin staining results also showed that the portion of Al accumulation in roots of *S. viridis* NT plants occurs in the DTZ (**Figure 3A**). Thus, it is worth to verify the gene expression profile of target genes related to Al tolerance in different portions of *S. viridis* roots in non-transformed or transgenic plants submitted to Al stress, an approach that is currently being performed by our group.

As described above, the exudation of organic acid anions by the root apex is a common mechanism to confer Al tolerance in plants, as these compounds are able to chelate this metal in the rhizosphere, avoiding root absorption. It was demonstrated that MATE homologs were root citrate transporters in different monocot species such as maize and rice (Maron et al., 2010, 2013; Yokosho et al., 2011). Here, it was demonstrated that a new MATE homolog, *BdMATE*, when overexpressed in *S. viridis* plants, was able to increase root citrate exudation in transgenic plants when compared to NT plants. We verified the exudation of citrate, the major organic acid anion secreted through the MATE transporter (Magalhães et al., 2007; Kochian et al., 2015) in *S. viridis* plants in the absence or presence of Al. Our results confirmed that transgenic plants overexpressing the *BdMATE* gene secreted more citrate than NT plants in both conditions (**Figure 3B**). Organic acids are metabolically active solutes with a diverse array of functions in many organisms. These acids have a potential role in the osmotic adjustment and in the balance of cation concentration of the cells, for instance. As described above, OAs anions are secreted by the root apex as an aluminum resistance mechanism in most plants. Citrate, malate, and oxalate are the main OAs anions secreted to the rhizosphere in many plant species (Kochian et al., 2015). Organic acids (OAs) are produced mainly in the mitochondria, by the TCA cycle and, in a lesser extent, in the glyoxysome (López-Bucio et al., 2000). The accumulation of OAs produced by TCA cycle is dependent upon the plant species, age of the plant and tissue type. In addition, the exudation of OAs anions by roots is dependent on the route of involvement of these OAs in specific physiological functions (Rees, 1990; López-Bucio et al., 2000). Recently, Nunes-Nesi et al. (2014) discussed the involvement of enzymes of the TCA cycle in response to the stress of aluminum, since OAs participate as key components in the mechanisms that some plants use to cope with nutritional deficiency and metal tolerance. Since citrate and malate are the main OAs anions secreted to the rhizosphere under Al stress, we aimed to investigate the expression levels of genes related to CS and MD, responsible for citrate and malate production, respectively, in the transgenic plants overexpressing *BdMATE*. It could be possible that the tolerance phenotype presented by transgenic plants under Al stress was due to an increased expression of genes responsible for the production of citrate or malate by these plants. However, we were not able to verify any difference in the gene expression levels between transgenic and NT plants, in the absence or presence of Al (**Figure 4**).

The relationship between increased CS activity and Al tolerance is still inconclusive. In some species such as *Triticum aestivum* L. (wheat) and *Secale cereale* L. (rye), the evaluation of CS production in aluminum stress conditions have been performed aluminum (Li et al., 2000). Plantlets of cv. King of the *S. cereale* demonstrated an increased CS activity when exposed to aluminum for more than 6 h, while in cv. Atlas 66 of the *T. aestivum* L. there was no difference in enzyme activity in plantlets subjected to aluminum (Ryan et al., 1993; Li et al., 2000). Our results demonstrated that *S. viridis* transgenic plants overexpressing *BdMATE* with Al tolerance phenotype do not increase the levels of CS gene expression. As mentioned above, the OAs that the plant uses in order to tolerate the aluminum through exudation by the root apex are metabolites originated in the TCA cycle, a key metabolic pathway to all organisms. Thus, it is plausible that the tolerance mechanism to aluminum in plants has a significant carbon cost, being highly regulated (Ryan et al., 1993; Sivaguru and Horst, 1998; Magalhães et al., 2007; Sivaguru et al., 2013). The CS gene is constantly being expressed to maintain the balance between citrate production and consumption by the cell. The product of the *CS* gene, citrate, is a molecule that is converted rapidly in the TCA cycle and/or can be degraded in other cellular compartments to ensure that the metabolic system may be constantly supplied (negative feedback). Thus, it could be very difficult to observe drastic changes in the expression levels of a gene that is involved in a fine-tune regulation of pivotal metabolic processes in the cell. Therefore, more studies are necessary in order to elucidate the relationship between the levels of citrate produced by the TCA cycle with Al tolerance in plants. We were not able to measure if the transformed plants had a carbon loss penalty compared to NT plants, especially because the experiments were performed in hydroponic conditions during a short time. We are currently establishing Al stress experiments in soil to further study the biomass of plants transformed with *BdMATE*. In addition, root-specific and/or Al-induced promoters are being currently studied by our group, to minimize the putative carbon loss caused by the constitutive expression of *BdMATE*.

Altogether, our results suggest that a C4 plant overexpressing a *B. distachyon MATE* transporter gene (*BdMATE*) was more tolerant to aluminum stress than wild plants through the exudation of citrate into the rhizosphere, with subsequent avoidance of Al absorption and accumulation in the DTZ of the roots. As consequence, the transgenic plants demonstrated sustainable root growth in the presence of Al when compared to NT plants. Our results suggested that, in the future, the overexpression of *BdMATE* could be used to develop Al tolerant crops closely related to *S. viridis* such as maize, wheat, and sugarcane.

## Author Contributions

HM, AK, AR, PM, WRS, and AA conceived and designed the experiments. PM and AR performed the *in silico* analysis. AR, WRS, FV, and KD performed the abiotic stress assays. AR, BC, and FV carried out the RT-qPCR assays and analyzed the data. AR, PM, WRS, and AK performed hematoxylin assay. PAO, RC, DC, WRS, MB, and HM performed and analyzed the organic acids. WRS and AR wrote the manuscript. AR, WRS, GC, JVM, CS, MB, and AA contributed to the discussion of the results. HM, AK, and GC provided intellectual input and revised the manuscript. All authors read and approved the final manuscript.

## Conflict of Interest Statement

The authors declare that the research was conducted in the absence of any commercial or financial relationships that could be construed as a potential conflict of interest.

## Footnotes

1 http://phytozome.jgi.doe.gov

## Abbreviations

Al^3+^: trivalent aluminum
CIM: callus induction media
CS: citrate synthase
DTZ: distal transition zone
MATE: multidrug and toxic compound extrusion
MD: malate dehydrogenase
NT: non-transgenic
RGI: root growth inhibition
RNG: root net growth
SRM: selective regeneration medium
TCA: tricarboxylic acid.
