# Supplementary material for: Overexpression of BdMATE Gene Improves Aluminum Tolerance in Setaria viridis
Source: Front Plant Sci. 2017 Jun 8;8:865. doi: 10.3389/fpls.2017.00865 (PMC5462932; doi:10.3389/fpls.2017.00865)
Supplement: Supplementary file 1 [file Table_1.doc]

Supplementary Material

# OVEREXPRESSION OF *BdMATE* GENE IMPROVES ALUMINUM TOLERANCE IN *Setaria viridis*

Ana Paula Ribeiro^1,2^, Wagner Rodrigo de Souza^1^, Polyana Kelly Martins^1^, Felipe Vinecky^1^, Karoline Estefani Duarte^1^, Marcos Fernando Basso^1^, Bárbara Andrade Dias Brito da Cunha^1^, Raquel Bombarda Campanha^1^, Patrícia Abrão de Oliveira^1^, Danilo da Cruz Centeno^3^, Geraldo Magela de Almeida Cançado^4^, Jurandir Vieira de Magalhães^5^, Carlos Antônio Ferreira de Souza^1^, Alan Carvalho Andrade^2,6^, Adilson Kenji Kobayashi^1^ and Hugo Bruno Correa Molinari^1*^

*** Correspondence:** Corresponding Author: hugo.molinari@embrapa.br

**Supplementary Table S1.** Sequence of the pair of primers used for PCR and qPCR analysis.

| Oligo Name | | Sequence (5’ → 3’) | Amplicon  Size | Annealing TºC |
| --- | --- | --- | --- | --- |
| *Bd*MATE | *F^*^* | GGAAGAAAACAGCAGCCAAG | 450 pb | 55 |
|  | *R^**^* | ATGTGGCAAACGAACATCAA |  |  |
| *Zm*Ubi1 | *F^*^* | CGAGTAGATAATGCCAGCCT | 456 pb | 58 |
|  | *R^**^* | GACGAGCGGCGTACCTTGAA |  |  |
| *hpt* | *F^*^* | TGTTTATCGGCACTTTGCAT | 511 pb | 56 |
|  | *R^**^* | GATGTTGGCGACCTCGTATT |  |  |
| *Si*SDHq | *F^*^* | TGTCGGCTCCTGTTGCAAA | 80 pb | 60 |
|  | *R^**^* | CGGTGGGAGATCGAGAATGT |  |  |
| *Si*UBCq | *F^*^* | TCTAGTTCGCCAGCAAAGCA |  |  |
|  | *R^**^* | CATCTGGTCTCCTTTGGCGA |  |  |
| *Bd*MATEq | *F^*^* | TCTAGTTCGCCAGCAAAGCA |  |  |
|  | *R^**^* | CATCTGGTCTCCTTTGGCGA |  |  |
| *Sv*MATEq | *F^*^* | ACTCTGCCTACTCCATGGTTGC |  |  |
|  | *R^**^* | AAAGGTACACCAGGCACGGTATG |  |  |
| *Si*EF1-αq | *F^*^* | TGGTATGCTTGTCACCTTTGGT |  |  |
|  | *R^**^* | CTCGTGGTGCATCTCAACTGA |  |  |
| *Si*SUIq | *F^*^* | CCAGAGCTTGGACAGGTCATTC |  |  |
|  | *R^**^* | ACAATGCCAGCCTGGACAA |  |  |
| *Si*CACq | *F^*^* | CTGCTTCTGGTCTTCGTGTT |  |  |
|  | *R^**^* | GTATGATCCTGCTCTCGTGATG |  |  |
| *Si*CULq | *F^*^* | TCTCATCACGAGGGACTACTT |  |  |
|  | *R^**^* | CTTGCCAACAACCACCAATC |  |  |
| *Si*CSq | *F^*^* | AGGGTCTCCTTTGGCTTCTTCT |  |  |
|  | *R^**^* | GGCCAGCAATTCCTTTGAAA |  |  |
| *Si*MDq | *F^*^* | CCAGGGACGACCTCTTCAAC |  |  |
|  | *R^**^* | AGCGTTTGGGCAGTGCTT |  |  |

**Forward*; ***Reverse*
