# Supplementary material for: Overexpression of BdMATE Gene Improves Aluminum Tolerance in Setaria viridis
Source: Front Plant Sci. 2017 Jun 8;8:865. doi: 10.3389/fpls.2017.00865 (PMC5462932; doi:10.3389/fpls.2017.00865)
Supplement: Supplementary file 2 [file Table_2.doc]

Supplementary Material

# OVEREXPRESSION OF *BdMATE* GENE IMPROVES ALUMINUM TOLERANCE IN *Setaria viridis*

Ana Paula Ribeiro^1,2^, Wagner Rodrigo de Souza^1^, Polyana Kelly Martins^1^, Felipe Vinecky^1^, Karoline Estefani Duarte^1^, Marcos Fernando Basso^1^, Bárbara Andrade Dias Brito da Cunha^1^, Raquel Bombarda Campanha^1^, Patrícia Abrão de Oliveira^1^, Danilo da Cruz Centeno^3^, Geraldo Magela de Almeida Cançado^4^, Jurandir Vieira de Magalhães^5^, Carlos Antônio Ferreira de Souza^1^, Alan Carvalho Andrade^2,6^, Adilson Kenji Kobayashi^1^ and Hugo Bruno Correa Molinari^1*^

*** Correspondence:** Corresponding Author: hugo.molinari@embrapa.br

**Supplementary Table S2.** Segregation ratios in the *T_1_* generation of plants transformed with *Zm*Ubi1::*Bd*MATE.

| **Event** | **Resistant ^*a^** | **Sensitive ^*b^** | **χ^2^ value for 3:1^*c^** | **Fits 3:1 ratio^*d^** |
| --- | --- | --- | --- | --- |
| 28 | 27 | 11 | 0.59 | Y |
| 29 | 27 | 3 | 3.60 | Y |
| 35 | 21 | 8 | 0.10 | Y |
| 91 | 34 | 19 | 3.32 | Y |
| 49 | 29 | 9 | 0.03 | Y |
| 39 | 30 | 8 | 0.31 | Y |
| 48 | 31 | 14 | 0.86 | Y |

^*a^ Number of seedlings survived in the medium containing 50 mg/L hygromicin B.

^*b^ Number of seedlings sensitive in the medium containing 50 mg/L hygromicin B.

^*c^ Significantly different at *P* < 0.05

^*d^ Ratio of hygromycin resistant:sensitive seedlings
