# Supplementary material for: Overexpression of BdMATE Gene Improves Aluminum Tolerance in Setaria viridis
Source: Front Plant Sci. 2017 Jun 8;8:865. doi: 10.3389/fpls.2017.00865 (PMC5462932; doi:10.3389/fpls.2017.00865)
Supplement: Supplementary file 3 [file Image_1.pdf]

Supplementary Material

OVEREXPRESSION OF *BdMATE* GENE IMPROVES ALUMINUM TOLERANCE IN *Setaria viridis*

Ana Paula Ribeiro[1,2], Wagner Rodrigo de Souza[1], Polyana Kelly Martins[1], Felipe Vinecky[1], Karoline Estefani Duarte[1], Marcos Fernando Basso[1], Bárbara Andrade Dias Brito da Cunha[1], Raquel Bombarda Campanha[1], Patrícia Abrão de Oliveira[1], Danilo da Cruz Centeno[3], Geraldo Magela de Almeida Cançado[4], Jurandir Vieira de Magalhães[5], Carlos Antônio Ferreira de Sousa[1], Alan Carvalho Andrade[2,6], Adilson Kenji Kobayashi[1] and Hugo Bruno Correa Molinari[1*]

*** Correspondence:** Corresponding Author: hugo.molinari@embrapa.br

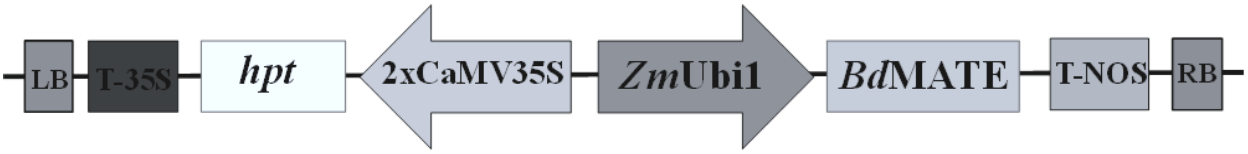

Supplementary Figure S1. Schematic representation of the binary vector *p6i* (DNA Cloning Service, Germany). The vector contains the *BdMATE* gene under the control of *ZmUbi1* promoter. The selective marker is *hpt* gene under the control of the 2x*CaMV35S* promoter, conferring resistance to hygromycin
