# Supplementary material for: Overexpression of BdMATE Gene Improves Aluminum Tolerance in Setaria viridis
Source: Front Plant Sci. 2017 Jun 8;8:865. doi: 10.3389/fpls.2017.00865 (PMC5462932; doi:10.3389/fpls.2017.00865)
Supplement: Supplementary file 4 [file Image_2.pdf]

Supplementary Material

OVEREXPRESSION OF *BdMATE* GENE IMPROVES ALUMINUM TOLERANCE IN *Setaria viridis*

Ana Paula Ribeiro[1,2], Wagner Rodrigo de Souza[1], Polyana Kelly Martins[1], Felipe Vinecky[1], Karoline Estefani Duarte[1], Marcos Fernando Basso[1], Bárbara Andrade Dias Brito da Cunha[1], Raquel Bombarda Campanha[1], Patrícia Abrão de Oliveira[1], Danilo da Cruz Centeno[3], Geraldo Magela de Almeida Cançado[4], Jurandir Vieira de Magalhães[5], Carlos Antônio Ferreira de Sousa[1], Alan Carvalho Andrade[2,6], Adilson Kenji Kobayashi[1] and Hugo Bruno Correa Molinari[1*]

*** Correspondence:** Corresponding Author: hugo.molinari@embrapa.br

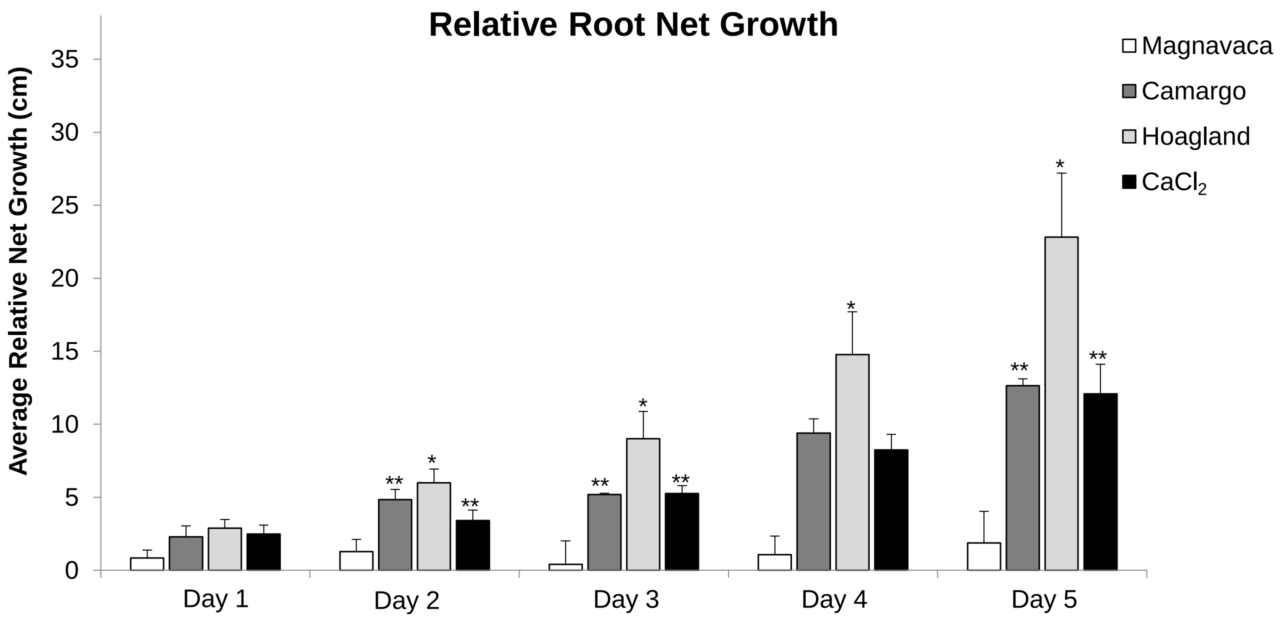

Supplementary Figure S2. Relative Root Net Growth of *Setaria viridis*. NT plants grown under the nutrient solution Magnavaca (Magnavaca et al., 1987), Camargo (Camargo, 1981), Hoagland (Hoagland and Arnon, 1950) and 500 µM $CaCl_2$ solution (Sasaki et al., 2004) during 1-5 days (n = 20 plantlets). *Significantly different at $P < 0.05$ Hoagland nutrient solution and others solutions. **No difference between Camargo and 500 µM $CaCl_2$ solution and statistically different between Magnavaca nutrient solution.
