# Supplementary material for: Overexpression of BdMATE Gene Improves Aluminum Tolerance in Setaria viridis
Source: Front Plant Sci. 2017 Jun 8;8:865. doi: 10.3389/fpls.2017.00865 (PMC5462932; doi:10.3389/fpls.2017.00865)
Supplement: Supplementary file 7 [file Image_5.pdf]

Supplementary Material

OVEREXPRESSION OF *BdMATE* GENE IMPROVES ALUMINUM TOLERANCE IN *Setaria viridis*

Ana Paula Ribeiro[1,2], Wagner Rodrigo de Souza[1], Polyana Kelly Martins[1], Felipe Vinecky[1], Karoline Estefani Duarte[1], Marcos Fernando Basso[1], Bárbara Andrade Dias Brito da Cunha[1], Raquel Bombarda Campanha[1], Patrícia Abrão de Oliveira[1], Danilo da Cruz Centeno[3], Geraldo Magela de Almeida Cançado[4], Jurandir Vieira de Magalhães[5], Carlos Antônio Ferreira de Sousa[1], Alan Carvalho Andrade[2,6], Adilson Kenji Kobayashi[1] and Hugo Bruno Correa Molinari[1*]

*** Correspondence:** Corresponding Author: hugo.molinari@embrapa.br

```
BdMATE(Bradig69770.1)      MEDG-A----------PTTMTQEKRLA----VVVVAPE-------DPAAAPVLLANGAAA
SbMATE(Sb03g043890)        MEEHRSPAHAKPEAEQPPQQQVPAAMAVAVAVDVAAPAALQNSTAAPAENGDVAAAGAAE
                           **:  :          *        :*    * *.**         **    : * ***

BdMATE(Bradig69770.1)      EEHKAGEDPPAPPTALLSGWPRRTGLYLFVMNVRSVFKLDELGSEVLRIAVPASLALAAD
SbMATE(Sb03g043890)        NGTAASAANGDGGGSELLGGPRWTGLHLFVMNIRSVFKLDELGAEVLGIAVPASLALTAD
                           :   *.        : * * ** ***:*****:**********:*** *********:**

BdMATE(Bradig69770.1)      PLASLVDTAFIGRLGSVEIAAVGVSIAIFNQVSKVCIYPLVSVTTSFVAEEDAIISKYLE
SbMATE(Sb03g043890)        PLASLIDTAFIGRLGSVEIAAVGVAIAVFNQVMKVCIYPLVSVTTSFVAEEDAVLSKGGA
                           *****:******************:**:**** ********************::**

BdMATE(Bradig69770.1)      ENSS-----QDLEKAPVDS-----------EASNVPVSG---AECVNSCIPTECT--DLS
SbMATE(Sb03g043890)        KVIDNGEEEEELEAGQVGPEKHTAAAGADPEKQQQPADEEAAKNGGEGCAPAVVAGRSSG
                           :  .     ::** . *.            * .: *..     :  :.* *:  :  . .

BdMATE(Bradig69770.1)      NQGCKRKYIPSVTSALIVGSFLGLVQAVFLIFSAKVVLGIMGVKHDSPMLEPAVRYLKIR
SbMATE(Sb03g043890)        KKSGNRRFVPSVTSALIVGALLGLFQTVFLVAAGKPLLRLMGVKPGSPMVMPALRYLTLR
                           ::. :*:::**********::***.*:***: :.* :* :**** .***: **:***.:*

BdMATE(Bradig69770.1)      SLGAPAVLLSLAMQGVFRGFKDTKTPLYATVVGDAANIILDPILMFVCHMGVTGAAVAHV
SbMATE(Sb03g043890)        ALGAPAVLLSLAMQGVFRGFKDAKTPLYAIVAGDAANIVLDPILIFGCRLGVIGAAIAHV
                           :*********************:****** *.******:*****:* *::** ***:***

BdMATE(Bradig69770.1)      VSQYLITMILICRLVQQVDVIPPSLKSLKFGRFLGCGFLLLARVVAVTFCVTLASSLAAR
SbMATE(Sb03g043890)        LSQYLITLIMLSKLVRKVDVVPPSLKCLKFRRFLGCGFLLLARVVAVTFCVTLAASLAAR
                           :******:*::.:**::***:*****.*** ***********************:*****

BdMATE(Bradig69770.1)      DGATIMAAFQICCQLWLATSLLADGLAVAGQAVLASAFAKNDNNKVVAATSRVLQLSIVL
SbMATE(Sb03g043890)        HGPTAMAAFQICTQVWLATSLLADGLAVAGQAMIASAFAKEDRYKVAATAARVLQLGVVL
                           .* * ******* *:*****************::******:*. **.*:::*****.:**

BdMATE(Bradig69770.1)      GMGLTVVLGLFMKFGAGVFTKDAAVIDVIHRGIPFVAGTQTINALAFVFDGINFGASDYT
SbMATE(Sb03g043890)        GAALTALLGLGLQFGAGVFTSDAAVIKTIRKGVPFVAGTQTLNTLAFVFDGINFGASDYA
                           * .**.:*** ::*******.*****..*::*:********:*:***************:

BdMATE(Bradig69770.1)      YSAYSMVGVAAISIPCLVYLSAHNGFIGIWVALTIYMSLRTIASTWRMGAARGPWVFLRK
SbMATE(Sb03g043890)        FSAYSMIGVAAVSIPSLIFLSSHGGFVGIWVALTIYMGVRALASTWRMAAAQGPWKFLRQ
                           :*****:****:***.*::**:*.**:**********.:*::******.**:*** ***:

BdMATE(Bradig69770.1)      GW
SbMATE(Sb03g043890)        --
```

Supplementary Figure S5. Alignment of the deduced amino acid sequences. Clustal Omega (Sievers and Higgins, 2014) alignment of the deduced amino acid sequences of B*rachypodium distachyon* (*Bd*MATE) and *Sorghum bicolor* (*Sb*MATE) sequences. Symbols under the alignments indicate identical (*), strongly conserved (:) or weakly conserved (.) residues according to the BLOSUM62 matrix.
