# Supplementary material for: Overexpression of BdMATE Gene Improves Aluminum Tolerance in Setaria viridis
Source: Front Plant Sci. 2017 Jun 8;8:865. doi: 10.3389/fpls.2017.00865 (PMC5462932; doi:10.3389/fpls.2017.00865)
Supplement: Supplementary file 10 [file Image_8.pdf]

Supplementary Material

OVEREXPRESSION OF *BdMATE* GENE IMPROVES ALUMINUM TOLERANCE IN *Setaria viridis*

Ana Paula Ribeiro[1,2], Wagner Rodrigo de Souza[1], Polyana Kelly Martins[1], Felipe Vinecky[1], Karoline Estefani Duarte[1], Marcos Fernando Basso[1], Bárbara Andrade Dias Brito da Cunha[1], Raquel Bombarda Campanha[1], Patrícia Abrão de Oliveira[1], Danilo da Cruz Centeno[3], Geraldo Magela de Almeida Cançado[4], Jurandir Vieira de Magalhães[5], Carlos Antônio Ferreira de Sousa[1], Alan Carvalho Andrade[2,6], Adilson Kenji Kobayashi[1] and Hugo Bruno Correa Molinari[1*]

*** Correspondence:** Corresponding Author: hugo.molinari@embrapa.br

```
BdMATE(Bradig69770.1)         MEDGAPTT---------MTQEKRLAVVVVAPEDPAAAPVLLANGAAAEEHKAGEDPPAPP
SvMATE(Sevir.9G493200.2)      MEDNNSAAAAASVVTTSVPAEKHVAI----P-AAAVAAMTNGGGSAAEESK-AEEDMLPP
                              ***.  ::         :  **::*:    *   *.* :  ..*:**** * .*:   **

BdMATE(Bradig69770.1)         TALLSGWPRRTGLYLFVMNVRSVFKLDELGSEVLRIAVPASLALAADPLASLVDTAFIGR
SvMATE(Sevir.9G493200.2)      APALPCGPRKTGLHLFIMNIRSVFKLDELGSEVLRIAVPASLALAADPLASLIDTAFIGR
                              :  *   **:***:**:**:********************************:*******

BdMATE(Bradig69770.1)         LGSVEIAAVGVSIAIFNQVSKVCIYPLVSVTTSFVAEEDAIISKYLEENSSQDLEKAP-V
SvMATE(Sevir.9G493200.2)      LGSVEIAAVGVSIAIFNQVSKVCIYPLVSVTTSFVAEEDAIISKAVEENSSHDLEKASHV
                              ******************************************** :*****:*****  *

BdMATE(Bradig69770.1)         DSEASNVPVSG---AECVNSCIPTECTDLSNQGCKRKYIPSVTSALIVGSFLGLVQAVFL
SvMATE(Sevir.9G493200.2)      DSETNNSPVSGPDIAECVNTCIPTECTELSNHGSKKKYIPSVTSALIVGSILGLLQAIFL
                              ***:.* ****   *****:*******:***:*.*:**************:***:**:**

BdMATE(Bradig69770.1)         IFSAKVVLGIMGVKHDSPMLEPAVRYLKIRSLGAPAVLLSLAMQGVFRGFKDTKTPLYAT
SvMATE(Sevir.9G493200.2)      VFSAKFVLSIMGVKSGSPMQGPAVRYLTIRSLGAPAVLLSLAMQGVFRGFKDTKTPLYAT
                              :****.**.***** .***  ******.********************************

BdMATE(Bradig69770.1)         VVGDAANIILDPILMFVCHMGVTGAAVAHVVSQYLITMILICRLVQQVDVIPPSLKSLKF
SvMATE(Sevir.9G493200.2)      VVGDATNIILDPILMFVCHMGVTGAAVAHVASQYLITLILLCRLVQQVDVIPPSIKSLKF
                              *****:************************.******:**:*************:*****

BdMATE(Bradig69770.1)         GRFLGCGFLLLARVVAVTFCVTLASSLAARDGATIMAAFQICCQLWLATSLLADGLAVAG
SvMATE(Sevir.9G493200.2)      GRFLGCGFLLLARVVAVTFCVTLAASLAARQGPTIMAGFQICCQLWLATSLLADGLAVAG
                              ************************:*****:* ****.**********************

BdMATE(Bradig69770.1)         QAVLASAFAKNDNNKVVAATSRVLQLSIVLGMGLTVVLGLFMKFGAGVFTKDAAVIDVIH
SvMATE(Sevir.9G493200.2)      QAVLASAFAKNDNKKVVAATSRVLQLSIVLGMCLTVVLGLAMKFGAGIFTKDLPVIEVIH
                              *************:****************** ******* ******:****  **:***

BdMATE(Bradig69770.1)         RGIPFVAGTQTINALAFVFDGINFGASDYTYSAYSMVGVAAISIPCLVYLSAHNGFIGIW
SvMATE(Sevir.9G493200.2)      KGIPFVAGTQTINSLAFVFDGINFGASDYTYSAYSMVAVASVSIPCLVYLSAHNGFIGIW
                              :************:***********************.**::******************

BdMATE(Bradig69770.1)         VALTIYMSLRTIASTWRMGAARGPWVFLRKGW
SvMATE(Sevir.9G493200.2)      IALTIYMSLRTIASTWRMGAARGPWTFLRK--
                              :************************.****
```

Supplementary Figure S8. Alignment of the deduced amino acid sequences. Clustal Omega (Sievers and Higgins, 2014) alignment of the deduced amino acid sequences of *Brachypodium distachyon* (*Bd*MATE) and *Setaria viridis* (*Sv*MATE) sequences. Symbols under the alignments indicate identical (*), strongly conserved (:) or weakly conserved (.) residues according to the BLOSUM62 matrix.
